# A Practical Manual of Diseases of the Skin

**Published:** 1892-09

**Authors:** 


A PRACTICAL MANUAL OF DISEASES OF THE SKIN.—By George H. Rohe, M. D , Professor of Materia Medica, Therapeutics and Hygiene, and formerly Professor of Dermatology in the College of Physicians and Surgeons, Baltimore, etc. Assisted by J. Williams Lord, A. B., M. D., Lecturer on Dermatology, etc., in the College of Physicians and Surgeons, Baltimore, etc. Published by the F. A. Davis Co., Philadelphia, Pa.

THIS little work of Dr. Rohe's is very creditable, and contains much that is valuable. The medicamentation mentioned throughout the book is very fine, and his classification and pathology good. H. H.

